# Driving in the Dark: Designing Autonomous Vehicles for Reducing Light Pollution

**DOI:** 10.1007/s11948-019-00101-7

**Published:** 2019-03-22

**Authors:** Taylor Stone, Filippo Santoni de Sio, Pieter E. Vermaas

**Affiliations:** 1 Department of Industrial Design, Delft University of Technology, Delft, Netherlands; 2 Department Ethics/Philosophy of Technology, Delft University of Technology, Delft, Netherlands

**Keywords:** Autonomous vehicles, Ethics of self-driving cars, Nighttime lighting, Light pollution, Transportation ethics, Responsible innovation of self-driving cars, Design for values

## Abstract

This paper proposes that autonomous vehicles should be designed to reduce light pollution. In support of this specific proposal, a moral assessment of autonomous vehicles more comprehensive than the dilemmatic life-and-death questions of trolley problem-style situations is presented. The paper therefore consists of two interrelated arguments. The first is that autonomous vehicles are currently still a technology in development, and not one that has acquired its definitive shape, meaning the design of both the vehicles and the surrounding infrastructure is open-ended. *Design for values* is utilized to articulate a path forward, by which engineering ethics should strive to incorporate values into a technology during its development phase. Second, it is argued that nighttime lighting—a critical supporting infrastructure—should be a *prima facie* consideration for autonomous vehicles during their development phase. It is shown that a reduction in light pollution, and more boldly a better balance of lighting and darkness, can be achieved via the design of future autonomous vehicles. Two case studies are examined (parking lots and highways) through which autonomous vehicles may be designed for “driving in the dark.” Nighttime lighting issues are thus inserted into a broader ethics of autonomous vehicles, while simultaneously introducing questions of autonomous vehicles into debates about light pollution.

## Introduction

Autonomous vehicles have the potential to revolutionize transportation networks and radically transform urban design strategies. Exactly what this future will look like, though, is still open for debate and scrutiny. Yet while visions of future cities and roadways dominated by “driverless cars” remain nebulous, their impending realization has garnered a growing technical and ethical debate.^1^ Current technical discourse largely focuses on the potential benefits in terms of safety, easing congestion, and emissions reductions (e.g., Hoogendoorn et al. 2014; Diakaki et al. 2015; Fagnant and Kockelman 2014, 2015). Research is also exploring the tangential effects of driving automation on issues such as vehicle ownership and sharing, land use, energy consumption, air pollution, and public health (e.g., Duarte and Ratti 2018; Milakis et al. 2017). Taking a more critical approach, ethical discourse has largely focused on how vehicles should be programmed to behave in dilemmatic life-and-death scenarios, and what decision-making criteria should be followed (e.g., Bonnefon et al. 2016; Gogoll and Müller 2017; Lin 2016; Santoni de Sio 2017). The issues under debate are then how these vehicles should be programmed to operate in such circumstances, who should decide on this programming, and where the resultant moral and legal responsibility lies.^2^

While important considerations, critiques have nevertheless been raised about this pathway for ethical discourse, including the over-reliance on viewing autonomous vehicles as a real-life manifestation of the “trolley problem” (JafariNaimi 2017; Nyholm and Smids 2016), the lack of attention to social justice issues (Epting 2018; Mladenovic and McPherson 2016), and the need for systems level analyses (Borenstein et al. 2017). These critiques highlight a broader issue with over-emphasizing hypothetical dilemmatic scenarios: they focus on a yet-to-be-realized endpoint, assuming that fully autonomous vehicles have been introduced into the existing physical, behavioural, and institutional landscape. Further, it leads the ethics of autonomous vehicles towards an ethics of collision programming. This risks overlooking the larger landscape of social and environmental challenges—and opportunities—this new technology may create, and the moral issues at stake therein.^3^

Given the potentially transformative impact of autonomous vehicles on a broad range of moral, social, and environmental values, there is an opportunity—and arguably a duty—to broaden ethical analyses and consider how (and why) to develop this technology. For this task a *design for values* approach is adopted, which asserts that societal and moral values should be proactively taken into account from the early stages of the design and development process, thus embedding values into the technical system (van den Hoven et al. 2015). Importantly, this approach allows for a questioning of basic presuppositions about both vehicle design *and* the surrounding infrastructure that this new technology will shape, and be shaped by (e.g., Heinrichs 2016; Milakis et al. 2017).

Such an approach necessitates ethical research into a range of issues related to the physical infrastructure, institutions, and socio-technical systems interwoven with transportation networks. This paper examines one specific topic in detail, namely the relationship between autonomous vehicles and streetlights, a critical piece of transportation infrastructure that has yet to receive significant attention. The adverse effects of artificial nighttime lighting—known as light pollution—have emerged as a pressing environmental issue, costing billions of dollars, using enormous amounts of energy, negatively affecting human health and ecosystems, and hindering experiences of a natural night sky (Stone 2017). To combat these effects, “[t]he challenge faced by 21st century policymakers is to provide outdoor light where and when it is needed while reducing costs, improving visibility, and minimizing any adverse effects on plants, animals, and humans caused through exposure to unnatural levels of light at night” (Kyba et al. 2014, p. 1807). The introduction of autonomous vehicles is a rare and pivotal opportunity to take up this challenge. Questions of light pollution could therefore be part of the landscape of values and goals influencing the development of autonomous vehicles and surrounding infrastructure. This paper offers a novel analysis of the confluence of two technologies with seemingly disparate moral challenges—autonomous vehicles and nighttime lighting—exploring how autonomous driving systems could be designed to reduce light pollution and create darker nights.

This paper will proceed as follows. A comprehensive ethics of autonomous vehicles, which utilizes a *design for values* approach to proactively incorporate ethical concerns into the predicted short-to-medium term development phases, is put forward to contextualize this paper. This is followed by a look into the ethics of nighttime lightning, and in particular the issue of light pollution. The *value of darkness* (Stone 2018b) is introduced as a moral framework for nighttime lighting, and applied to road lighting. In doing so, a weak and strong moral claim is articulated. At the least, autonomous vehicles must minimize the negative effects and costs of light pollution. Yet they can also go further, striving to actively promote the valuableness of darkness and help to re-imagine urban nightscapes. Following this bolder position, two scenarios are sketched in which lighting infrastructure can be adapted for “driving in the dark”—parking lots and highways—as are the design requirements this places on future high-automation vehicles.

## Towards a Comprehensive Ethics of Autonomous Vehicles

To examine the ethical issue of how to design autonomous driving systems for reducing light pollution and realizing a better balance between lighting and darkness, the following principles are followed (adapted from Filippo Santoni de Sio 2016):

- Focusing on the process towards full automation and the full range of possible varieties of (partial) autonomy rather than only on one hypothetical fully-autonomous (“driverless”) scenario;
- Going beyond collision programming and towards the design of the entire socio-technical system, including technical infrastructures, social and legal norms, and educational systems;
- Broadening the scope of possible ethical issues involved in the design of future systems—not only risks for life and physical integrity, but also justice, privacy, inclusion, environment, etc.; and,
- Taking a proactive approach and considering how ethical trade-offs (moral dilemmas) can be solved through design, by relying on a value-sensitive approach

Before turning to light pollution and presenting possible “driving in the dark” scenarios, two of these principles require further explanation: varieties of automation (a) and a proactive design for values approach (d).

### Varieties of Automation

Ethical debates often focus on “driverless” or “self-driving” cars—in other words, fully autonomous vehicles. However, such debates often jump to a hypothetical endpoint of both technical development as well as social and institutional adoption of this new technology. Thus, these are simplifications that a comprehensive ethical approach—with an attention to the full range of values at stake in the development of technology, *as well as* maintaining relevance to the real world of technology and policy—cannot afford. Therefore, before engaging in any ethical reflection on autonomous vehicles we should be clear on at least two issues: what different levels of automation are possible, and, what reasonable timelines for their adoption would be.^4^

According to a standard taxonomy, SAE International standard J3016 (SAE 2016), vehicle autonomy ranges from 0—no automation, to 5—full automation, with the autonomous driving system controlling all aspects and modes of driving. A key distinction in the taxonomy is between level 2 and 3, when the autonomous system takes over an entire “dynamic driving task.” However, at level 3 the human driver still has a responsibility to intervene at the request of the system. In levels 4 and 5—“high automation” and “full automation,” respectively—this is no longer the case. Levels 4 and 5 are also called higher-order automation, insofar as “the driver no longer has to monitor the vehicle or system continuously” (Beiker 2016, p. 194). However, a critical difference is that whereas in level 5 the vehicle can drive autonomously under all scenarios—mixed traffic, city centers, highways, parking, high and low speed roads, etc.—at level 4 vehicles can only drive without human supervision in specific scenarios, for instance highways and parking lots.

Ethical literature focused on dilemmatic scenarios typically take level 5 vehicles as a given—“driverless cars” operating in mixed traffic scenarios and interacting with different sorts of road users (e.g., non-autonomous vehicles, cyclists, pedestrians) in various driving scenarios (highways, urban roads, country roads). However, notwithstanding the recurrent claims in the media that driverless cars “are coming,” and although some in the car industry cite 2020 as a target date for fully autonomous vehicles, scientific researchers tend to be more cautious. An expert and enthusiast pioneer in vehicle automation like Steven Shladover (2016) is sceptical that full automation (level 5) will happen any time before 2075; however he believes that level 4 as defined by SAE (full automation for limited tasks) will likely be possible in the next decade. For example, he believes that autonomous valet parking and autonomous freeway systems (which form the basis of the two case studies included later on) will be realities within 10 years. However, once the technology is available, there are still questions regarding the rate of consumer adoption, as well as necessary policy and institutional changes. According to Sven Beiker (2016), in a scenario of continuous technological (and market) evolution, it would likely still take at least 15–20 years for there to be a significant share of cars in operation with higher-order automation (even though more niche-based innovations like autonomous taxis might take hold more quickly).^5^ The Netherlands Institute for Transport Policy Analysis (KiM) predicts a similar trajectory, expecting a full transition to high-automation occurring around 2060–2100 (KiM 2015). Based on these predictions, this paper assumes that while full automation (level 5) is not likely to happen on a large scale in the near future, automation under limited conditions (level 4) is likely to be achieved and in use within the next 15–20 years. A comprehensive ethics of autonomous vehicles should therefore investigate the opportunities (and risks) that less-than-fully autonomous vehicles may bring, as well as anticipate issues that might arise during the transition period towards higher-level automation.

### Steering the Future: Design for Values

Accepting that the future of autonomous vehicles is open, and that different scenarios for development and adoption can unfold, this future becomes one influenced by the choices of actors within all kinds of technical and social processes, including industry, governance, economics, and politics. Rather than retroactively observing how these choices and processes are eventually realized in specific scenarios, social and environmental values can guide a process of proactively creating scenarios that comply with these goals. From an engineering perspective, including values in the design process may seem counterintuitive, since engineering design has traditionally viewed new products or technologies as value-neutral, developed only on the basis of functional requirements. However, from the perspective of (consumer) product development, and fields such as architecture and fashion design, values are standard elements that co-shape design processes. For example, cars are already designed not only for enabling transportation at a specific speed, but also for expressing personality, style, wealth, masculinity, etc. Likewise, various requirements for a wide range of engineered products and services, such as safety and sustainability, are in fact value-laden concepts deeply embedded into the design process.

There are roughly two ways in which social and environmental values can be injected into the design of technologies. The first is to take identified values as *constraints* to design. Designers should actively explore whether the new product or technology could violate or come into conflict with the values of stakeholders. If so, designers should adjust the design of the product or technology such that these conflicts are avoided. The *value*-*sensitive design* method developed by Batya Friedman and colleagues (2006) follows in part this more precautionary, constraint-oriented approach.

Alternatively, social and environmental values can be articulated as requirements within the design process, alongside functional requirements. In this way, values are not only constraints against which designs should be checked, but also targets that immediately co-define the product or technology under development. For example, the design of a new bridge in a city can be seen as a project aimed at meeting functional requirements, such as allowing specific traffic flows, as well as at realising values such as expressing the innovative character of the city, or inclusiveness by allowing pedestrians and cyclists to use it. This more integrated approach is developed under the heading of *design for values* (van den Hoven 2007, 2013; van den Hoven et al. 2015^6^).

The later “driving in the dark” scenarios primarily adopt the latter design for values approach. The openness of the development of autonomous driving systems, combined with their potential to fundamentally transform transportation networks, creates a unique and pivotal opportunity to include social and environmental values as design criteria. Importantly, such an approach leads to the question: *what values should be incorporated into the design of autonomous vehicles and surrounding infrastructure*?

## The Function and Morality of Nighttime Lighting

Within this more comprehensive ethics of autonomous vehicles, a careful articulation, and justification, of values worth pursuing becomes an important task. Here, the focus is on one particular set of values (and one particular technological domain) that has yet to receive critical attention, but for which the introduction of high-automation vehicles introduces important possibilities: nighttime lighting.^7^ A recent editorial in *Lighting Research & Technology* hints at the impending impact of autonomous vehicles by highlighting the existential crisis facing streetlights:

It is predicted that by 2040 most vehicles sold will be autonomous. This raises an interesting question. If there is no driver who needs to see the way ahead, is the rationale for providing much road lighting gone? The potential represented by these impending technologies suggests to me that now would be a good time for all those involved in road lighting to ask themselves some fundamental questions. What is the purpose of road lighting? If it is no longer necessary to allow drivers to see where they are going, what is it for? (Boyce 2016, p. 787)

While a critique of the timeline proposed by Peter Boyce was put forward above, as well as a clarification of what sorts of autonomous tasks may soon be realized, this call to action nevertheless signals a need to elucidate the values informing the “fundamental questions” driving the future need and function of streetlights. This means extending technical and moral discussions of autonomous vehicles to include the impacts of (transportation-related) nighttime lighting, and vice versa.

### Light Pollution and the Value of Darkness

One lighting-related concern is the adverse effects of artificial illumination at night, known as *light pollution*. The concept of light pollution was popularized in the 1970s to describe and categorize the negative effects of artificial nighttime lighting, and has since emerged as an important environmental concern for the 21st century (Stone 2017). Terrel Gallaway (2010, p. 72) defines light pollution as “the unintended consequences of poorly designed and injudiciously used artificial lighting.” In the USA, approximately 30% of outdoor lighting is considered to be “wasted,” estimated to cost upwards of 7 billion US dollars per year. Furthermore, eliminating this excess lighting could have the same reduction in CO_2_ emissions as removing ~ 9.5 million cars from the road (Gallaway et al. 2010). An estimate of the excess and wasted nighttime lighting in the European Union puts the costs at over 5 billion Euros per year (Morgan-Taylor 2014). In addition to the financial costs and energy usage, artificial lighting at night has negative effects on human health, as well as wildlife and ecosystems (e.g., Gaston et al. 2015; Longcore and Rich 2004; Pottharst and Könecke 2013). And, ever-present *skyglow*, perhaps the most pervasive effect of light pollution around urbanized regions, is increasingly cutting off access to a starry night sky—experiences that arguably carry significant cultural value (e.g., Bogard 2013; Gallaway 2014). It is estimated that over 80% the world, and more than 99% in Europe and North America, now live in regions with “polluted” night skies (Falchi et al. 2016).

Acknowledging that light pollution is an important issue in its own right, and intertwined with larger societal concerns (e.g., sustainability and climate change), it can be argued that there is a moral obligation to work towards eliminating, or at least mitigating, the above adverse effects. Existing efforts to curb the light pollution include ordinances at local, national, and even trans-national levels, with goals of emissions reductions, energy (and cost) efficiency, and in some cases dark sky protection.^8^ There are also efforts focused on proper technical standards for lighting fixtures, colour temperature, and brightness (e.g., IDA-IES 2011). And, in recent years “dark sky reserve” programs have emerged, aimed at the protection and conservation of unpolluted night skies in wilderness areas or national parks (Meier 2014). Such efforts are important and have led to successes in both curbing light pollution and raising public awareness. However, much of the developed world continues to get brighter, and this trend is expected to further increase with the widespread adoption of LED streetlights (Falchi et al. 2016; Kyba et al. 2017). Continued efforts are therefore needed, including proposals for more radical or transformative changes. We must consider longer-range ideas to effectively “design out” many of the causes of light pollution in ways that are, in the formulation of Ibo van de Poel (2016), both morally acceptable and socially accepted; that is, that can reduce negative effects without hindering the desirable and necessary aspects of nighttime illumination.

In efforts to seek out more radical or transformative strategies to nighttime lighting, it is useful to also seek out new moral frameworks—to elucidate underlying judgments and re-frame the problem at hand. Shaping concerns about light pollution is an important shift in how we perceive and evaluate darkness at night. Historically seen as evil, chaotic, and dangerous, darkness is increasingly seen as something of positive environmental and cultural value (Bogard 2013; Edensor 2017). To understand how darkness could be viewed as something beneficial for urban nightscapes, the framework of Taylor Stone (2018b) is utilized here. The commonly recognized effects of light pollution are re-framed as nine ways by which, or through which, value is derived from darkness. From these nine values, *prima facie* obligations are derived as principles to be considered in the design of nightscapes, even if not achievable in every case (Table 1). This provides a comprehensive set of design goals for nighttime lighting that incorporates environmental values, thus going beyond only mitigating “polluting” effects. A focus on the value of darkness can therefore allow for a re-evaluation of *all* nighttime lighting, ultimately offering more drastic energy and cost savings. Importantly, this framework does not rest on a total de-valuing of lighting at night, but rather on an appreciation of natural nighttime conditions and the potential created by an attentive and restrained use of artificial lighting.

**Table 1:** Prima facie obligations to consider in the design of nighttime lighting; for a more detailed description of these values, as well as their interrelations and prioritization, see Stone 2018b, pp. 614–623

Value of darkness	Prima facie obligation derived from value
Efficiency	The responsible use of lighting where and when needed; money-saving
Sustainability	The responsible use of lighting where and when needed; energy-saving and preserving non-renewable resources
Ecological conservation	The protection and preservation of species and biodiversity; habitat conservation efforts
Healthiness	Promoting and fostering human health; physiological well-being
Happiness	Promoting and fostering happiness; emotional well-being
Connection to nature	Preserving a connection to the more-than-human world
Stellar visibility	Preserving conditions for access to the firmament
Heritage and tradition	Preserving the cultural heritage of the night sky for future generations
Wonder and beauty	Preserving the aesthetic appeal of the natural night sky

### Designing for Darkness

Street lighting and vehicle lights combine to create one of the largest sources of illumination at night, and therefore should be seen as a pressing environmental issue (Lyytimäki et al. 2012). One needs only to view an aerial photo (at the scale of cities, nations, or even continents) to observe the presence of transportation-related lighting, as illuminated grids and lines carving through the landscape. According to the International Energy Agency (2006), globally there are more than 100 million streetlights, using approximately 114 TWh of electricity annually. Parking lots are responsible for an estimated 55 million additional lights in OECD countries alone, consuming an additional 88 TWh of electricity in 2005. Taken together, street and parking lot lighting combine to constitute over 90% of outdoor illumination (International Energy Agency 2006). In the European Union, lighting accounts for 14% of total energy consumption; of that, approximately 14.7% is outdoor stationary lighting, which are mainly streetlights. Globally, almost one-fifth of all electricity produced is used for lighting, of which approximately 8% is outdoor stationary lighting (De Almeida et al. 2014). Another area of impact is the vehicle headlights. It is estimated that each year over 55 billion litres of gasoline or diesel is used to operate vehicle lights, equating to about 3.2% of total vehicle fuel use, and equivalent to the consumption of over 1 million barrels of oil daily (International Energy Agency 2006).

Design strategies that address nighttime lighting can take the form of a weak or strong position. First, future autonomous vehicles must, at the very least, strive to *reduce* the adverse effects and costs caused by transportation-related illumination. Given the ties to efficiency and sustainability (cost savings, GHG reductions, etc.), as well as likely health and ecosystem benefits, there is no moral justification for omitting consideration of this design requirement. The degree to which light pollution can be reduced, and if this may compromise other desired goals, are additional questions outside the scope of this paper. For instance, a position often taken is that nighttime lighting increases safety and should therefore be extended rather than be reduced.^9^ Acknowledging the importance of such questions, however, should not block the adoption of light pollution concerns as a *prima facie* requirement in the development of autonomous driving systems.

A second, stronger claim, although more bold and visionary, can be derived from a design for values approach: future autonomous vehicles and surrounding infrastructure should actively *promote* the value of darkness. The transformative potential of higher-automation vehicles offers an opportunity to fundamentally re-consider how (and why) to light our nightscapes. The vehicle-focused lighting strategies of the 20^th^ century can be replaced with alternative approaches, which actively strive to bring some darkness back into urban nightscapes. The effects can be far reaching, ranging from lighting that is more attentive to pedestrian and cycling traffic, to more intimate and convivial urban spaces, to ecologically-oriented “dark design” (Edensor 2017), to re-envisioning ideas of the nocturnal sublime within urbanized areas (Stone 2018a). As mentioned above, such an approach does not imply a goal of eliminating *all* nighttime lighting, but a better balance of light and dark that is attentive to functional needs and environmental values. In sum, autonomous vehicles can work towards achieving what Tim Edensor (2015, p. 436) poetically describes as a “re-enchantment of the night” via a conscientious re-introduction of urban darkness.

## Realizing Darkness with Autonomous Vehicles

If the bolder position articulated above is adopted, how would this steer future innovation? What scenarios and related design requirements would eliminate much of the need for transportation-focused streetlights, thus allowing for a drastic reduction in light pollution and a conscientious re-imagining of (urban) darkness? What does this mean for the design choices for autonomous vehicles themselves, as well as surrounding infrastructure and institutions? Such questions are complex, requiring technical, moral, legal, policy, and design work for a full answer. Here first steps are taken by providing a preliminary sketch of what such a path forward would entail.

Accepting the timeline of technology development and adoption as earlier laid out, it can be expected that level 4 automation—where the system has full control for limited tasks—will be available within the next decade, and market saturation may occur over the coming 20–30 years. These tasks, though limited, provide a testbed for the viability of “driving in the dark” scenarios, and represent “low hanging fruit” for immediate positive effects. With this in mind, two scenarios that are candidates for full automation in the near future are introduced: parking lots and highways. These build on the similar scenarios proposed by Walther Wachenfeld and colleagues (2016), adding the potential for substantial, and relatively immediate, positive impact towards the creation of darker nights. Both have a singular functionality and are primarily used by vehicles, thus avoiding issues such as pedestrian and cyclist interactions. Equally important, their lighting is singularly focused on vehicle usage, with little or no ancillary benefits (aesthetic, social, etc.), meaning that a drastic reduction would have minimal impact on other nearby types and uses of illumination or nighttime activities. Following these two case studies, the resultant design requirements for the autonomous vehicles themselves are briefly considered.

### Scenario 1: Parking in the Dark

The first scenario builds on the use case “Autonomous Valet Parking” described by Wachenfeld and colleagues (2016, pp. 14–16). As the name indicates, the autonomous system acts as a personal valet. One exits the vehicle at a destination, inputs a nearby parking lot for the system, and the vehicle parks itself. Similarly, a pick up location would be chosen (similar to ride-sharing services) and the vehicle would come pick you up. Such a scenario typically means a short driving distance for the autonomous program (and in cases such as shopping malls, driving only within the parking lot itself), low speeds, and lighter traffic. Hence, this can be seen as an introductory scenario of level-4 automation for (personally-owned) vehicles.

The value-add proposed here is that parking lots designated for autonomous valet parking no longer require constant illumination. This would be cost-saving for the lot owner and reduce energy consumption. And, it would greatly decrease light pollution, especially if this could be introduced in suburban areas around shopping malls etc., where parking lots take up extensive space. With only contingency lighting in place for maintenance, security, and emergencies, the parking lots could be left in the dark. The nature of parking lots also makes the incremental rollout of “parking in the dark” possible—specific lots or sections can be converted gradually, based on demand. Thus in the short term designated darkened parking lots can be introduced, with this trend spreading if autonomous parking becomes the norm in future generations of vehicles, *and* if dark parking gains support and public acceptance.

Overall, this scenario is seen as having high impact potential—recall that globally there are more than 55 million lights used for parking lots (International Energy Agency 2006). Furthermore, it can be applied in a variety of settings—commercial areas, urban downtowns, suburban and residential areas, etc.—allowing for a wide distribution of benefits. It may create new concerns about crime, although dark campus programs have reportedly seen reductions in vandalism through reduced nighttime illumination (Henderson 2010). Even so, it would still necessitate new protocols for security, as well as safety considerations regarding barriers to entry or some form of technology-supported surveillance such as infrared cameras or alarm sensors (especially in areas where pedestrian traffic is close by).

### Scenario 2: Dark Highways

The second scenario builds on the use case described by Wachenfeld and colleagues (2016, pp. 12–14) of “Interstate Pilot Using Driver for Extended Availability.” In this scenario, once the vehicle has entered the highway the driver can—or must—activate the robot and relinquish driving responsibilities. After a destination is entered, the autonomous system will take over navigation, guidance, and control of the vehicle. At the pre-determined off-ramp or exit, the autonomous system coordinates a safe handover, with backup emergency procedures if the driver is unresponsive. Important to note is that “highways” here is used to describe a broader typology of roadways, which are given different names in different (social and use) contexts: freeways, interstates, expressways, etc. However, the common characteristics of these roadways are most important. First, they are used exclusively for high-speed vehicle traffic. Second, access is only possible by special connecting elements, such as on/off ramps (Wachenfeld et al. 2016). This means they will be devoid of pedestrians and cyclists, as well as intersections. Despite their high speeds, the simple surroundings, driving tasks, and minimal “dynamic objects” means that this can be considered as an introductory use case for autonomous systems (Wachenfeld et al. 2016).

Similar to the scenario above, the value-add is that highways would no longer require lighting, save for on/off ramps and emergency situations. While promising, the adoption of darkened highways presents more complications than the dark parking scenario above. First, it would require that *all* vehicles on the road use autonomous systems; so long as one car has a human driver, all lights are required. Thus, a high level of market saturation, combined with regulatory changes to vehicle requirements, would be required. A second issue would be user acceptance, as this would be a somewhat radical change in driving habits. One can imagine some initial hesitation to being a passenger in an autonomous vehicle travelling 120 km/hr with no lights above, no headlights, and no brake lights! Yet these concerns, though certainly well grounded, are not insurmountable. There has been widespread adoption of train and airplane travel, where passengers have eventually come to simply rely on the system to safely bring them to destinations, even if they cannot control or even see what is happening in front of the vehicle. Thus, such concerns do not make darkened highways immediately untenable as an ambitious medium-term goal, and the potential reduction to nighttime lighting offered by such scenarios is great enough to warrant further investigation. Certainly, in line with a design for values approach, this initial *normative* proposal should be integrated with empirical research into users’ behaviour and responses.

### Design Requirements for Dark-Driving Autonomous Vehicles

For these and other possible *driving in the dark* scenarios, a final consideration is the development of autonomous vehicles themselves. When adopting the value(s) of darkness as a design goal, it may seem obvious that efforts towards their realization should directly focus on the brightly lit and extensive road systems, and in a subsidiary way to adaptations of the vehicles for which the roads are meant. Yet, a simultaneous focus on the re-design of the vehicles is also required. For example, efficiency in car lighting is beneficial to increasing the distance cars can drive, establishing a link to the instrumental values of efficiency and sustainability—something particularly relevant for the introduction of electric vehicles. Also in this respect, it should be emphasized that here we are making general *normative* proposals for what should be integrated into the technical and empirical work on autonomous driving systems. Thus, these should simply be understood as initial considerations to be assessed, and if possible integrated, via future technical research.

Towards the goal of designing autonomous vehicles to function without both streetlights *and* headlights, a few key requirements can be identified (Table 2). First, it requires a “higher-order” level of automation—level 4 or higher as per the SAE taxonomy (SAE 2016). This will need to be accompanied by social and institutional changes, for it requires some consideration of when (or if) this technology should be “grandfathered” into new vehicles by laws and regulations, and a timeline for turning off lights in parking lots and especially on highways. Both scenarios would also require a re-design of transition zones, as well as new safety and emergency protocols. Another important consideration is the development of sensor technologies and navigation systems. The “driving in the dark” scenarios require a continuous investment in systems that require little or no lighting to navigate at night, such as on LiDAR (“light detecting and ranging”) technology coupled with maps, GPS, etc. This can potentially allow autonomous systems to drive in total darkness, as evidenced in an early test by Ford (e.g., Burgess 2016; Korosec 2016). Designing for low-light navigation will undoubtedly raise new technical challenges, including how to detect traffic signs and lane markings, as well as how to detect unexpected objects such as debris or wildlife—an especially important issue for highway safety. Further, the required technologies may be financially prohibitive in their current form. But again, this does not detract from the assertion that this *should* be one explicit design goal during the current development phase, in order to explore what possibilities are technically, financially, and socially achievable, and at what scale.

**Table 2:** General design considerations for autonomous driving systems to operate in the proposed “driving in the dark” scenarios

Vehicle feature	Design considerations
Level of automation	Requires the development and adoption of “higher-order automation”—level 4 or higher as per SAE taxonomy
Sensor technologies	Requires a continuous investment in sensor technologies that require low or no lighting to navigate at night, such as LiDAR (“light detecting and ranging”) technology, coupled with maps, GPS, etc.
Associated social/institutional changes	Requires some consideration of if or when this technology should be “grandfathered” into new vehicles by law, and a timeline for turning off lights in parking lots and on highways; both would require a re-design of transition zones and safety protocols

A more general design consideration is the new user experiences that can be offered by driving in the dark. Cars are often framed as means to give people freedom and access to natural settings, which can hypothetically find its way into the design of cars through features like panoramic transparent roofs, allowing passengers to enjoy natural landscapes and nightscapes. Switching off the lights would substantially increase experiences of the wonder and beauty of the starry night sky, further fostering the intrinsic goods of darkness (see Stone 2018a, b; Table 1).

## Conclusion

This paper proposed that the development of autonomous vehicles should incorporate, among other things, the ethics of nighttime lighting. At the least, autonomous vehicles should be designed to reduce the adverse effects of light pollution. More radically, they can strive to create darker nights and play a role in re-imagining urban nightscapes. To frame such a proposal, an ethics of autonomous driving systems broader than the dilemmatic life-and-death questions of trolley problem-style situations is required. A *design for values* approach to engineering ethics, in which values are pro-actively incorporated into technologies during their development phase, opens up a range of potential issues that can—and arguably should—be addressed in both the design of autonomous vehicles and their surrounding physical and institutional infrastructure.

The scenarios for dark parking lots and highways presented in this paper should not be seen as definitive, but are rather starting points for incorporating the ethics of nighttime lighting into a broader ethics of autonomous driving systems. And considered otherwise, it shows how autonomous vehicles, as one example of an emerging technology with profound transformative and disruptive potential, can be inserted into discourse on nighttime lighting. While the development of future roadways may not necessarily adopt these (or similar) scenarios in full, they must at least take this as a *prima facie* consideration in the development process. Future research should address the technical and financial feasibility of these scenarios, as well as study the possible social and psychological dimensions of their implementation.

Future research should also consider what new ethical problems could arise if these proposals are adopted. Langdon Winner (1980) famously showed the embedded-ness of politics in infrastructure by arguing that New York highway overpasses were explicitly made too low for public buses. This prevented public transportation from reaching certain locales, with the goal of hindering access to racial minorities and people of lower socio-economic status. One could similarly imagine that the proposals discussed in this paper inadvertently contribute to a similar scenario, where affluent areas or access roads are pro-actively darkened, therefore requiring high-automation vehicles and potentially limiting access based on socio-economic status. Hence, if “driving in the dark” scenarios are to be adopted, the landscape of potential ethical and political impacts must be continually explored alongside technological innovation. What is now needed is an iterative process for if (or how) the value of darkness gets incorporated into autonomous driving systems, and how it fits into the broader landscape of values at stake.

If the general argument is accepted, a follow-up question is then what other social and environmental issues should be considered during the development phase of autonomous vehicles. This requires a careful consideration of both institutional (e.g., ownership models) and physical (e.g., the design of mixed-use urban centers) infrastructures, as well as new issues created by autonomous systems, such as data security. And, this could lead to a re-design of various services that make use of vehicles (e.g., ambulances, garbage pick-up, or package deliveries). Put more bluntly, ethicists must continue to critically and creatively explore what a future of “driverless cars” can, and should, entail.

## Footnotes

1 In this paper “autonomous vehicles” and “autonomous driving systems” are used interchangeably. However, this is meant to be a broader categorization than “driverless cars” or “self-driving cars,” which refers to a specific level of (high) automation and a specific use and function of the automated systems. Thus, these latter terms represent a set of assumptions that this paper is critical towards.

2 With the exception of Robert Sparrow and Mark Howard (2017) who reflect on the moral obligation to realize the transition to a fully autonomous transport system (assuming that this will be safer).

3 For example, a recent review paper by Fábio Duarte and Carlo Ratti (2018) explores the ways in which autonomous vehicles may impact cities and urban design, including issues such as parking, urban sprawl, and traffic congestion. All of these impacts have ethical dimensions that need to be examined in tandem with technical development.

4 Other factors to be included in a comprehensive analysis would be ownership and business models (see ITF Roundtable 2016).

5 Beiker (2016) bases this prediction on the timeline of adoption for other new features introduced in the past, such as anti-lock braking systems, and that vehicle fleets are typically replaced over a 20-year period.

6 The *Handbook of Ethics, Values, and Technological Design* (van den Hoven et al. 2015), is an edited volume featuring several contributions spanning theoretical debates, specific values, and application domains.

7 It is worth noting that the focus on light pollution is not meant to suggest that this is the only, or the most important or urgent, issue to be pursued by the design of future autonomous vehicles. Rather, it is *one* facet, among others, for which autonomous driving systems can and should be designed, and thus taken as a *prima facie* consideration.

8 Christopher Kyba and colleagues (2014) provide an overview of existing ordinances aimed at curbing light pollution.

9 The relationship between lighting and safety (both perceived and actual) is outside the scope of this paper, but for overviews of the debate see for example Gaston et al. (2015) and Pottharst and Könecke (2013).
